# Impact du traitement antirétroviral sur le profil biologique des enfants VIH positifs suivis au Centre Hospitalier et Universitaire de Yaoundé au Cameroun

**DOI:** 10.11604/pamj.2015.20.159.4677

**Published:** 2015-02-19

**Authors:** Ginette Claude Mireille Kalla, Marie-Claire Okomo Assoumou, Nelly Kamgaing, Francisca Monebenimp, Francois-Xavier Mbopi-Keou

**Affiliations:** 1 Faculté de Médecine et des Sciences Biomédicales, Université de Yaoundé I, Yaoundé, Cameroun; 2 Centre Hospitalier Universitaire, Service de Pédiatrie, Yaoundé, Cameroun; 3 Ministère de la Santé Publique, Yaoundé, Cameroun

**Keywords:** Traitement antirétroviral, profil biologique des enfants VIH positifs, Cameroun, antiretroviral treatment, biological profile of HIV positive children, Cameroon

## Abstract

**Introduction:**

L'objectif de ce travail était d’évaluer l'impact du traitement antirétroviral sur le profil biologique des enfants VIH positifs suivis au Centre Hospitalier et Universitaire de Yaoundé au Cameroun.

**Méthodes:**

Il s'agissait d'une étude rétrospective réalisée de Mai 2003 à Décembre 2012 au CHU de Yaoundé au Cameroun. Pour cette étude, nous avons obtenu une clairance éthique.

**Résultats:**

L’âge moyen était de 54.02±46.34 mois. The sexe ratio était de 0.96 en faveur des garçons. Le diagnostic s’était fait tardivement (74.2%) ainsi que la mise sous traitement (83.3%). Seuls 36 des 116 enfants (31%) avait pu avoir un bilan biologique à l'initiation du traitement antirétroviral et six mois après l'initiation du traitement antirétroviral. Après six mois de traitement, nous avons enregistrés une augmentation significative des paramètres biologiques suivants: taux de glycémie de 0.09g/L (0.75-0.84; p= 0.007), pourcentage de CD4 chez les enfants de moins de 5 ans de 4.62% (20.12-24.75; p = 0.022), valeur absolue de CD4 chez les enfants de plus de 5 ans de 294 cellules/mm3 (151.18-445.18; p = 0.011), le rapport CD4/CD8 de 0.35 (0.55-0.90; p = 0.000). Enfin, après six mois de traitement, on enregistrait une baisse significative de la charge virale du VIH de 3.90 log (5.85-1.95; p = 0.006).

**Conclusion:**

Il ressort de cette étude que la restauration immunitaire et la suppression virologique peuvent être obtenus après six mois de traitement antirétroviral. Cependant, des efforts doivent encore être faits en ce qui concerne la prise en charge du suivi biologique, gage d'un bon suivi thérapeutique au Cameroun.

## Introduction

La pandémie de l'infection par le Virus de l'Immunodéficience Humaine (VIH) est loin d’être contrôlée et ce, en dépit des grandes interventions menées à l’échelle planétaire. L′Afrique subsaharienne abrite plus de 67% de toutes les Personnes Vivant avec le VIH (PVVIH), soit 22,5 millions [1]. L′accès au traitement antirétroviral (TAR) en Afrique subsaharienne reste très faible en raison d′obstacles [2–4] tels que le nombre limité de médecins, la mesure de la charge virale limitée, la problématique de la disponibilité d′examens plus simples comme la mesure des lymphocytes T CD4 ou les examens de biochimie [1]. Devant ces difficultés, l′Organisation Mondiale de la Santé (OMS) a proposé une approche de prise en charge «allégée» pour favoriser l′accès aux antirétroviraux (ARV) à grande échelle dans les pays à ressources limitées [5]. Les premiers programmes africains structurés d′accès aux ARV ont vu le jour en 1998 dans des pays comme le Sénégal, la Côte d′Ivoire et l′Ouganda [6–8]. Ces programmes ont montré l′efficacité du TAR en Afrique, avec des résultats comparables à ceux obtenus dans les pays du Nord en termes de survie, d′efficacité virologique, immunologique et clinique, d′observance, d′émergence des résistances et de toxicité [9, 10].

Dès l'apparition des premiers cas de SIDA en 1985 au Cameroun [11], divers plans de lutte contre le VIH/SIDA ont été élaborés et mis en oeuvre avec plus ou moins de succès de 1987 à nos jours [12]. Des progrès encourageants méritent d’être inscrits tels que l'accroissement important du nombre total des Centres de Traitement Agréés (CTA) et des Unités de Prise En charge du VIH/SIDA (UPEC), la gratuité des ARV depuis mai 2007, l'augmentation du nombre de personnes séropositives sous ARV [13]. Le Comité National de Lutte contre le SIDA (CNLS) rapporte en Décembre 2009 que sur 560 000 PVVIH, le Cameroun compterait près de 74.710 patients sous antirétroviraux (ARV), soit 39% des 153.185 PVVIH éligibles aux traitements dans l'ensemble du pays [14–16].

Au regard de toutes ces améliorations dans la prise en charge des PVVIH, nous nous sommes proposés d’évaluer l'impact du traitement antirétroviral sur le profil biologique des enfants VIH positifs suivis au Centre Hospitalier et Universitaire de Yaoundé au Cameroun.

## Méthodes

Il s'agissait d'une étude rétrospective réalisée de Mai 2003 à Décembre 2012 au Service de Pédiatrie du Centre Hospitalier Universitaire de Yaoundé (CHUY) au Cameroun.

**Population d’étude:** étaient retenus dans notre étude, tout enfant séropositif sous traitement antirétroviral. Cette étude a fait l'objet d'une clairance éthique.

**Recueil des données:** après avoir obtenu l'autorisation de consulter les archives, nous avons répertorié tous les dossiers médicaux des enfants VIH positifs, sous traitement antirétroviral suivis dans le Service de Pédiatrie du CHUY au Cameroun sur la période allant de 2003 à 2012. Le questionnaire utilisé s'intéressait aux caractéristiques sociodémographiques du patient, aux éléments de diagnostic, aux bilans biologiques à l'initiation du TAR et au sixième mois après le début du TAR.

**Analyse des données:** les données collectées ont été saisies et analysées par des méthodes de statistiques descriptives et analytiques en utilisant le logiciel SPSS 19. Les différences entre les proportions ont été analysées en utilisant des tables de contingence et en appliquant le test de Chi-2. Les valeurs p < 0,05 étaient considérées comme statistiquement significatives.

## Résultats

Nous avons recruté 116 enfants infectés par le VIH et remplissant les critères d'inclusion. Cinquante neuf enfants étaient de sexe masculin (50.9%) et cinquante sept de sexe féminin (49.1) soit un sexe ratio de 0.96 en faveur des garçons (Tableau 1). L’âge moyen était de 54.02±46.34 mois. La majorité de la population d’étude résidait en zone urbaine (84.5%) (Tableau 1). Tous les enfants de l’étude étaient VIH positifs (100%). Cependant, dans 76.7% de cas, le diagnostic de l'infection par le VIH était sérologique et dans 23,3% des cas, la confirmation avait été effectuée par la technique dite de polymérisation en chaine (PCR).

**Tableau 1:** Caractéristiques sociodémographiques de la population d’étude

Variables		Fréquence (n =116)	Pourcentage (%)
**Sexe**	Fille	57	49,1
	Garçon	59	50,9
**Age (mois)**	< 12	24	20,7
	12 - 35	27	23,3
	36 -59	22	19
	> 59	43	37
**Lieu de résidence**	Rural	18	15,5
	Urbain	98	84,5

La majorité des malades étaient à un stade clinique sévère (74,2%). Le déficit immunitaire était avancé dans 17,8% des cas et sévère chez 64,5% des malades (Tableau 2).

**Tableau 2:** Répartition de la population d’étude selon le diagnostic de l'infection à VIH

Variables		Fréquence (n = 116)	Pourcentage (%)
Méthode de dépistage	PCR	27	23,3
	Sérologie	89	76,7
Type de VIH	Inconnu	49	42,2
	VIH 1	67	57,8
	VIH 2	0	0
Stade clinique	OMS 1	2	1,7
	OMS 2	28	24,1
	OMS 3	73	62,9
	OMS 4	13	11,3
Déficit immunitaire	Pas de déficit	9	8,4
	Modéré	10	9,3
	Avancé	19	17,8
	Sévère	69	64,5

Seuls 36 des 116 enfants (31%) avait pu avoir un bilan biologique à l'initiation du traitement antirétroviral et six mois après l'initiation du traitement antirétroviral (Tableau 3). Après six mois de traitement, nous avons enregistrés une augmentation significative des paramètres biologiques suivants: taux de glycémie de 0.09g/L (0.75-0.84; p= 0.007), pourcentage de CD4 chez les enfants de moins de 5 ans de 4.62% (20.12-24.75; p = 0.022), valeur absolue de CD4 chez les enfants de plus de 5 ans de 294 cellules/mm3 (151.18-445.18; p = 0.011), le rapport CD4/CD8 de 0.35 (0.55-0.90; p = 0.000). Enfin, après six mois de traitement, on enregistrait une baisse significative de la charge virale du VIH de l'ordre de 3.90 log (5.85-1.95; p = 0.006) (Tableau 3).

**Tableau 3:** Comparaison des valeurs biologiques moyennes des 36 malades ayant fait le bilan biologique à l'initiation du traitement antirétroviral et six mois après le traitement antirétroviral

Variables		Taux moyen M 0	Taux moyen M 6	Différence	Valeur P	IC
Hb (g/dl)		9,71	10,55	0,83	0,081	(0,11 ; 1,78)
Glycémie (g/l)		0,75	0,84	0,09	0,007	(0,02 ; 0,15)
SGOT (UI/l)		43,76	53,64	9,88	0,459	(17,19 ; 36,95)
SGPT (UI/l)		28,24	43,28	15,04	0,460	(26,30 ; 56,38)
CD4	< 59 mois (%)	20,12	24,75	4,62	0,022	(0,74 ; 8,50)
	> 59 mois (cellules/mm^3^)	151,18	445,18	294	0,011	(83,41 ; 504,65)
CD8 (%)		40,10	33,95	6,14	0,009	(1,74 ; 10,54)
CD8 (cellules/mm^3^)		1215,88	1286,08	70,19	0,658	(252,65;393,03)
CD4/CD8		0,55	0,90	0,35	0,000	(0,19 ; 0,51)
CV (log _10_ copies/ml)		5,85	1,95	3,90	0,006	(2,15 ; 5,65)

## Discussion

Notre échantillon était de 116 malades. Cet effectif est inférieur à la majorité des études faites sur le suivi biologique des enfants VIH positifs sous TAR respectivement 477, 670, 790 et 151 enfants [1, 17–19]. Cependant, il est supérieur à l'effectif obtenu par Okomo et collaborateurs en Gambie qui a étudié une cohorte de 65 malades [18]. Par contre, il est proche de l'effectif de Musoke et collaborateurs réalisée en Ouganda en 2010 (n= 124) [20].

L’âge moyen était de 54 mois dans notre étude. Notre résultat est proche de ceux d'Ahoua et collaborateurs réalisée en Ouganda [21], Musoke et collaborateurs [20], Okomo et collaborateurs [18] dont l’âge moyen était respectivement de 60 mois, 60 mois et 58 mois. Il est inférieur à ceux de Janssen et collaborateurs réalisée dans le KwaZulu-Natal en Afrique du Sud [17], Isaakidis et collaborateurs au Cambodge [22], Edmonds et collaborateurs à Kinshasa en [23], Reddi et collaborateurs en Afrique du Sud [24] et Hansudewechakul et collaborateurs [25] dans une étude faite en Asie qui ont trouvé respectivement un âge moyen de 74 mois, 72 mois, 70 mois, 68 mois et 84 mois. Ceci montre bien que le diagnostic se fait tardivement dans les pays à ressources limitées.

Dans notre étude, nous avons eu 50,9% de garçon et 49,1% de fille. Le pourcentage de fille obtenu dans notre série est similaire à celui d'Isaakidis et collaborateurs (48%) [22] et Musoke et collaborateurs (49%) [20]. Il est supérieur à celui d'Ahoua et collaborateurs (45%) [21], et inférieur au résultat de Reddi et collaborateurs (51%) [24]. Concernant les garçons, notre résultat est presque identique à celui de Hansudewechakul et collaborateurs (50,4%) [25].

La majorité des malades dans notre série ont consulté à un stade clinique avancé soit (74,2%). Ces résultats se rapprochent de ceux obtenus par Janssen et collaborateurs (76,6%) [17], Ahoua et collaborateurs (75,6%) [21], Reddi et collaborateurs (70,2%) [24]. Okomo et collaborateurs [18], Edmonds et collaborateurs [23], et Hansudewechakul et collaborateurs [25] ont trouvé des taux inférieurs aux notres respectivement 41,5%, 51,3% et 50,2%. Le déficit immunitaire était sévère dans 82,3% de cas. Van Dijk et collaborateurs dans une étude réalisée en Zambie trouve dans les deux groupes qu'il a étudié respectivement un déficit immunitaire de 73,7% et 60,9% [26]. Edmonds et collaborateurs à Kinshasa trouve un déficit sévère dans 66,8% de cas [23].

Trente six malades ont faits le bilan biologique à l'initiation (M0) du TAR et six mois (M6) après le TAR. Nous avons comparé les valeurs biologiques moyennes de ces malades à M0 et M6 afin de voir si sous TAR il y avait une amélioration sur le plan biologique. Nous avons constaté une augmentation du taux moyen de toutes les variables étudiées avec une différence significative. Nous données se rapprochent de ceux de Van Dijk et collaborateurs, qui observe également dans sa série une augmentation du taux moyen des CD4 à M6 de 13% par rapport au taux moyen à l'initiation (respectivement 16,3% à M0 et 29,3% à M6) [26]. Okomo et collaborateurs trouve globalement un taux moyen de CD4 à M0 de 13% et à M6 de 23,3%, cette différence étant statistiquement significativement [18]. Nos résultats corrobent également ceux de Reddi et collaborateurs, qui, dans leur étude, observent une augmentation du taux moyen de CD4 de 7,8% à M0 à 18% à M6 [24].

## Conclusion

Il ressort de notre étude que la restauration immunitaire et la suppression virologique peuvent être obtenus après six mois de traitement antirétroviral. Notre étude mais surtout en exergue l'urgente nécessité d'une prise en charge du suivi biologique de l'infection par le VIH-notamment chez les personnes les plus vulnérables à l'instar des enfants- gage d'un bon suivi thérapeutique au Cameroun.

## References

[1] ONUSIDA (Programme commun des Nations Unies sur le VIH/SIDA) (2013). Rapport sur l’épidémie mondiale de SIDA 2013.

[2] Gruenais ME (2001). Un système de santé en mutation: le cas du Cameroun. Association Euro-Africaine pour l'Anthropologie du Changement Social et du Développement.

[3] McCoy D, Chopra M, Loewenson R, Aitken JM, Ngulube T (2005). Expanding access to antiretroviral therapy in sub-saharan Africa: avoiding the pitfalls and dangers, capitalizing on the opportunities. Am J Public Health..

[4] Schneider H, Blaauw D, Gilson L, Chabikuli N, Goudge J (2006). Health systems and access to antiretroviral drugs for HIV in Southern Africa: service delivery and human resources challenges. Reprod Health Matters..

[5] Gilks CF, Crowley S, Ekpini R, Gove S, Perriens J, Souteyrand Y (2006). WHO public-health approach to antiretroviral treatment against HIV in resource-limited settings. Lancet..

[6] Anonyme (1998). Le point sur la stratégie d'accessibilité et de disponibilité des antirétroviraux au Sénégal.

[7] Msellati P, Vidal L, Moatti JP (2000). Chronologie de l'Initiative Onusida Ministère de la Santé Publique d'accès aux traitements antirétroviraux pour les personnes vivant avec le VIH/sida en Côte-d'Ivoire. L'accès aux traitements du VIH/sida en Côte d'Ivoire.

[8] Coulaud JP (1997). Initiative internationale: place des antirétroviraux dans la prise en charge des personnes infectées par le VIH en Afrique.

[9] Desclaux A (1998). Évaluation et accompagnement de la multithérapie antirétrovirale chez les patients VIH-l du Sénégal. Aspects sociaux et observance.

[10] Seyler C, Anglaret X, Dakoury-Dogbo N, Messou E, Touré S, Danel C (2003). Medium-term survival, morbidity and immunovirological evolution in HIV-infected adults receiving antiretroviral therapy, Abidjan, Côte d'Ivoire. Antivir Ther..

[11] Mbopi-Kéou FX, Mpoudi-Ngolle E, Nkengasong J, Zekeng L, Mbanya D, Affana G (1998). Trends of AIDS epidemic in Cameroon, 1986 through 1995. J Acquir Immune Defic Syndr Hum Retrovirol..

[12] Plan stratégique national de lutte contre le VIH/SIDA 2006-2010.

[13] Eboko F, Abé C, Laurent C (2010). Accès décentralisé au traitement du VIH/sida: Evaluation de l'expérience camerounaise. Sciences sociales et SIDA..

[14] Rapport de progrès N° 11-septembre 2008: Vers l'accès universel aux traitements et soins en faveur des adultes et enfants vivant avec le VIH/SIDA au Cameroun.

[15] (2010). L'impact du VIH et du sida au Cameroun à l'horizon 2020.

[16] Boyer S, Blanche J, Marcellin F, Eboko F, Bonono CR, Ongolo-Zogo P (2008). Characteristics and early outcomes of HIV treatment decentralisation in Cameroon: results from the EVAL ANRS 12-116 study.

[17] Janssen N, Ndirangu J, Newel M-L, Bland RM (2010). Successful paediatric HIV treatment in rural primary care in Africa. Arch Dis Child..

[18] Okomo U, Togun T, Oko F, Peterson K, Townend J, Peterson I, Jaye A (2012). Treatment outcomes among HIV-1 and HIV-2 infected children initiating antiretroviral therapy in a concentrated low prevalence setting in West Africa. BMC Pediatr..

[19] Peacock-Villada E, Richardson BA, John-Stewart GC (2011). Post-HAART outcomes in pediatric populations: Comparison of resource limited and developed countries. Pediatrics.

[20] Musoke PM, Mudiope P, Barlow-Mosha LN, Ajuna P, Bagenda D, Mubiru MM, Tylleskar T, Fowler MG (2010). Growth, immune and viral responses in HIV infected African children receiving highly active antiretroviral therapy: a prospective cohort study. BMC Pediatr..

[21] Ahoua L, Guenther G, Rouzioux C, Pinoges L, Anguzu P, Taburet A-M, Balkan S, Olson DM, Olaro C, Pujades-Rodriguez M (2011). Immunovirological response to combines antiretroviral therapy and drug resistance patterns in children: 1- and 2-year outcomes in rural Uganda. BMC Pediatr..

[22] Isaakidis P, Raguenaud M-E, Te V, Tray CS, Akao K, Kumar V, Ngin S, Nerrienet E, Zachariah R (2010). High survival and treatment success sustained after two and three years of first line ART for children in Cambodia. J Int AIDS Soc..

[23] Edmonds A, Yotebieng M, Lusiama J, Matumona Y, Kitetele F, Napravnik S, Cole SR (2011). The effect of highly active antiretroviral therapy on the survival of HIV-infected children in a resource-deprived setting: A cohort study. Plos Med..

[24] Reddi A, Leeper SC, Grobler AC, Geddes R, France KH, Dorse GL, Vlok WJ (2007). Preliminary outcomes of paediatric highly active antiretroviral therapy cohort from KwaZulu-Natal, South Africa. BMC Pediatrics..

[25] Hansudewechakul R, Sirisanthana V, Kurniati N, Puthanakit T, Lumbiganon P, Saphonn V, Yusoff NK (2010). Antiretroviral therapy outcomes of HIV-infected children in the treat Asia pediatric HIV observational database. J Acquir Immune Defic Syndr..

[26] Van Dijk JH, Sutcliffe CG, Munsanje B, Sinywimaanzi P, Hamangaba F, Thuma PE, Moss WJ (2011). HIV-Infected children in rural Zambia achieve good immunologic and virologic outcomes two years after initiating antiretroviral therapy. Plos One..

